# Acceptability, Perceptions, and Experiences Regarding Electronic Patient-Reported Outcomes After Laparoscopic Cholecystectomy: Protocol for a Mixed Methods Feasibility Study

**DOI:** 10.2196/57344

**Published:** 2024-08-19

**Authors:** Kareem Choucair, Mark Corrigan, Adrian O'Sullivan, Sean Barber, Lucja Stankiewicz, Patrick Henn, Oscar Dennehy, Mohd Yasser Kayyal, Yong Yu Tan, Kayode Philip Fadahunsi, John O'Donoghue

**Affiliations:** 1 School of Medicine University College Cork Cork City Ireland; 2 Assert Research Centre University College Cork Cork City Ireland; 3 Malawi eHealth Research Centre University College Cork Cork City Ireland; 4 Cork University Hospital Cork City Ireland; 5 Mercy University Hospital Cork City Ireland; 6 Department of Primary Care and Public Health School of Public Health Imperial College London London United Kingdom; 7 Business Information Systems University College Cork Cork Ireland

**Keywords:** patient-reported outcomes, digital technology, hepatobiliary surgery, surgery, laparoscopic cholecystectomy, electronic patient, general surgeon, mixed methods, prospective study, quantitative, qualitative, Qualtrics, interview, Microsoft Teams, data collection, patient care, patient-centric, patient-doctor communication, eHealth

## Abstract

**Background:**

Patient-reported outcomes (PROs) can be defined as any report of a patient’s health taken directly from the patient. Routine collection of PRO data has been shown to offer potential benefits to patient-doctor communication. Electronic forms of PRO measures (PROMs) could be more beneficial in comparison to traditional PROMs in obtaining PROs from patients. However, it is currently unclear whether the routine collection of electronic PRO data could result in better outcomes for patients undergoing laparoscopic cholecystectomy (LC).

**Objective:**

This study aims to explore the perspectives of patients and surgeons on the use of electronic PROMs. Based on prior research, technical skill and experience level of the surgeon, long-term quality of life, patient involvement in decision-making, communication skills of the surgeon, cleanliness of the ward environment, and standards of nursing care are identified to be the most important factors for the patients.

**Methods:**

This is a mixed methods prospective study that will collect both quantitative (survey) and qualitative (interview) data. The study has two components. The first involves the distribution of an electronic presurvey to patients who received elective LC within 48 hours of their surgery (n=80). This survey will explore the perspective of patients regarding the procedure, hospital experience, long-term outcomes, and the perceived value of using PROMs. These patients will then be followed up after 1 year and given another survey. The second component involves the distribution of the same survey and the completion of structured interviews with general surgeons (n=10). The survey will ascertain what PROs from the participants are most useful for the surgeons and the interviews will focus on how the surgeons view routine PRO collection. A convenience sampling approach will be used. Surveys will be distributed through Qualtrics and interviews will be completed on Microsoft Teams.

**Results:**

Data collection began on February 14, 2023. As of February 12, 2024, 71 of 80 recruited patients have been given the presurvey. The follow-up with the patients and the general surgeon components of the study have not begun. The expected completion date of this study is in April 2025.

**Conclusions:**

Overall, this study will investigate the potential of electronic PRO collection to offer value for patients and general surgeons. This approach will ensure that patient care is investigated in a multifaceted way, offering patient-centric guidance to surgeons in their approach to care.

**International Registered Report Identifier (IRRID):**

DERR1-10.2196/57344

## Introduction

### Patient-Reported Outcomes

Patient-reported outcomes (PROs) can be defined as any report of a patient’s health outcome taken directly from the patient [1]. PROs are normally investigated through the use of standardized patient-reported outcome measures (PROMs), which are typically self-completed questionnaires that measure patient quality of life, symptom burden, functional status, health-related quality of life, and personal care experience [2,3]. The investigation of PROs allows for the generation of a health care system that is truly patient-centered [4]. Patient-centered health care has already proven to improve health status and the efficiency at which care is delivered [5]. Thus, it would be beneficial to understand the measures that could be taken to improve PROs after the completion of surgical procedures such as laparoscopic cystectomy (LC). PROs are particularly relevant to LCs since patients undergo this operation to relieve their pain; thus, measuring PROs after the procedure is crucial [6].

A systematic review demonstrated that most studies evaluating the effects of LC did not use an electronic method of measuring PROs, despite advances in technology [7]. Another systematic review concluded that there is limited literature regarding the use of electronic PRO systems in surgery, and the authors proposed that future research should explore the challenges associated with using such systems [8]. A more recent systematic review found that the integration of electronic PROM systems through the use of mobile apps has the potential to improve patient outcomes and the efficiency of hospital resources [9].

Han et al [6] demonstrated that LC results improved PROs regarding pain, quality of life, and the relief of gastrointestinal symptoms. Several factors significantly influence PROs after LC. For instance, a 2019 study in Singapore found that the technical skill and experience level of the surgeon, long-term quality of life, patient involvement in decision-making, communication skills of the surgeon, cleanliness of the ward environment, and standards of nursing care were crucial factors that influence PROs [10]. Conversely, factors such as hospitalization leave duration, length of hospital stay, the opinion of the patient’s family about the hospital, and scar cosmesis were factors with relatively less importance on PROs [10].

### Patient-Reported Experiences

Patient-reported experience measures (PREMs) are tools used to report on a patient’s process of care (eg, communication and timeliness of care) rather than on the outcomes specifically [2,11]. PREMs are a useful predictor for overall patient satisfaction [12]. As a result, PREMs will also be used in this study to evaluate the hospital experience of the patient.

### Routine Collection of PROs in Other Specialties

The implementation of routine PRO collection has been shown to be beneficial and feasible in various health care fields such as oncology, cardiology, and integrative medicine [13-16]. However, the integration of routine electronic PRO collection in oncology is currently limited due to inadequate information technology infrastructures, time, and difficulty in using electronic devices [17]. In the field of ophthalmology, one pilot study indicated that ophthalmologists recognized the powerful potential benefits of electronic PROs for patient-doctor communication [18]. Furthermore, an observational study [19] investigated whether passive collection of behavioral data could be associated with PROs using patients’ smartphone sensors and activity data, demonstrating that patients with lower activity levels tended to have poorer PRO scores. While the passive monitoring of PROs is beyond the scope of this project, this previous work highlights the immense potential benefits that routine PRO monitoring could have for LC and other general patients.

In the field of surgery, systematic reviews have indicated that routine PRO collection has benefits in relation to colorectal and orthopedic surgical specialties [20,21]. One quality improvement study also indicated that routine PRO collection was a critical factor in improving thoracic surgery care [22]. Although the logistical implication of routine collection remains the most challenging factor, the advancement of technology could assist in solving this issue. Thus, electronic PROMs could be useful for patients undergoing LC, which will be explored in this study.

### Routine Collection of PROs

The evidence that PROs can be useful as a feedback tool in surgery to improve patient outcomes is currently limited. One study indicated that there is currently only weak evidence that PROs provide any benefit to patient outcomes when used as a feedback tool [23]. Furthermore, more qualitative research is needed to investigate the practical issues that could arise with routine PRO collection [23,24]. However, another systematic review exploring routine PRO monitoring during cancer care demonstrated significantly improved health-related quality of life among patients [25]. These conflicts demonstrate a lack of clear consensus regarding the routine collection of PROs. Thus, this study will investigate the perspective of surgeons on PRO collection. These data could elucidate the type of feedback that is most relevant for surgeons to improve care. In addition, qualitative interviews will be conducted with the surgeons to obtain insight into the potential benefits and barriers of routine PRO collection.

The use of routine PRO collection specifically in relation to LC is currently very limited. One feasibility study investigating the use of electronic PROMs indicated that the collection of such measures could be a convenient process for patients that would be beneficial in evaluating quality-of-life trends for physicians [26]. Authors have also proposed the need for future research with more robust methodologies such as randomized controlled trials exploring this area. Using electronic PROMs has been indicated to help resolve the logistical and cost issues associated with PROM collection [13,26].

### Study Objectives

This study aims to explore the perspectives of patients and surgeons on the use of electronic PROMs. These perspectives will be investigated through the distribution of surveys to both groups and via the completion of interviews with the general surgeons.

The specific objectives of the study include (1) understanding the acceptability and perceived value of electronic PROs from the perspective of both patients undergoing LC and general surgeons and (2) determining the feasibility of following up with patients regarding their PROs using email correspondence.

## Methods

### Study Design and Overview

This is a mixed methods prospective study that will collect both qualitative and quantitative data. The study will involve two components. The first is the distribution of a survey to patients who have completed an elective LC. The second smaller component is the distribution of the same survey to consultant surgeons in addition to the completion of structured qualitative interviews with the same surgeons. This is an observational study and will have no influence on the care of patients.

### Component 1: Patient Survey

#### Patient Participants

The subjects will include all patients who completed elective LCs from Cork University Hospital and Mercy University Hospital, both in Cork, Ireland. Patients undergoing LC were chosen for this study owing to the high number of LCs completed in these hospitals. This makes the study more feasible and realistic to complete.

#### Inclusion Criteria

Patients must be >18 years old and have completed an elective LC. Patients must have the capacity to access the Qualtrics survey through their smartphone and have completed the LC within 48 hours of distribution of the first survey. For the sake of feasibility, any indication for the elective LC will be eligible for recruitment in this study to ensure an adequate sample size.

#### Exclusion Criteria

Patients who cannot read, are pregnant, have a mental disability, have dementia, or do not speak English will be excluded. Additionally, patients who are sedated, ventilated, or intubated will be excluded as they will not be capable of offering informed consent.

#### Sample Size

This study will aim to recruit 80 patients. This sample size was chosen for the sake of feasibility. Due to time constraints, with 10 months of allocated time for data collection and 2 participating hospitals collaborating on the project, it would not be feasible to collect more data.

Power calculation was considered for determining the sample size. However, power calculation requires an estimation of the proportion of the event of interest [27]. For this study, it would be unrealistic to estimate the number of elective LCs that are completed in Ireland, as these data are not readily available. Consequently, it was decided to estimate the sample size primarily based on feasibility rather than power.

#### Recruitment of Patient Participants

Participants will be recruited using a nonprobability convenience sampling approach. The patients undergoing elective LCs will be viewed from the daily theater list and through communication with the operating surgical team*.* Participating patients will be identified as eligible by surgeons from Cork University Hospital who are collaborating in this study. The participant information sheet and consent form will be integrated into the beginning of the Qualtrics survey.

After completion of the presurvey, the participants will be sent a link for the postsurvey 12 months later through email. The recruitment and data collection flow for patients is presented in Multimedia Appendix 1.

#### Patient Participant Survey

The survey will be based on the PROM questionnaire, which measures the effect of different factors on the PROs [10]. The following factors will be measured using the PROM survey: (1) age, sex, education level, marital status, employment status, ethnicity, smoking, and exercise frequency; (2) procedure perception; (3) hospital experience perception (PREMs); and (4) perception of longer-term consequences.

In addition, the acceptability and perceived value of the PROMs will be assessed with a survey from Stover et al [28].

The presurvey will be administered electronically by email using Qualtrics to patients who have completed an elective LC within the past 48 hours. These patients will then be followed up after 1 year and the survey will be administered again (Multimedia Appendix 2).

#### Other Data Collection Tools Considered

Other PRO surveys such as the Gastrointestinal Quality of Life Index survey and visual analog pain scores were also considered [29,30]. However, we ultimately determined to not use these tools in this study, as they only measure the PROs directly rather than determining what PROs and experiences patients find most important to them. Including a combination of these surveys could have been optimal, but it would have resulted in an overly long survey and potentially poor participant completion rates.

### Component 2: Surgeon Survey and Interview

#### Surgeon Population

Ten general surgeons will be recruited from Cork University Hospital and Mercy University Hospital. This sample size was chosen based on the feasibility within these hospital settings.

#### Inclusion Criteria

Only general surgeons will be included, as they represent the group that would be most knowledgeable about LC, which is the surgery that the study is based around. The surgeons must have been registrars, specialist registrars, or consultants for at least 1 year in a general surgery team. They must be currently employed within Cork University Hospital or Mercy University Hospital.

#### Exclusion Criteria

The surgeons involved in the creation of this study will be excluded from participation.

#### Recruitment of Surgeon Participants

The participants will be recruited using a nonprobability consecutive sampling approach. They will be briefed on the study using an information sheet and given a consent form for the interview.

#### Interview

All the surgeon participants (n=10) will also be interviewed using an interview guide adapted from the PROFILE trial [24]. The interviews will take place online using Microsoft Teams. The interviews will explore the following factors: (1) background of PRO use by the surgeon, (2) attitude of the general surgeon toward PRO use, (3) perception of the surgeons regarding the impact that PROs can have on clinical practice, and (4) perception of the surgeons regarding practical issues regarding PRO collection (Multimedia Appendix 3 and Multimedia Appendix 4).

#### Surgeon Survey

A modified version of the PROM questionnaire will be distributed to general surgeons (n=10). The general surgeons will be asked to complete the survey only once. The survey will be given to the surgeons with the goal of ascertaining what feedback from patients is most important for them to improve their quality of care. The modified survey will only measure the following factors: (1) procedure perception, (2) hospital perception, and (3) perception of longer-term consequences (see Multimedia Appendix 5).

#### Data Analysis

SPSS v28.0.1.1 (IBM Corp) will be used to complete the data analysis for the patient surveys. The 4-point Likert-scale questions will be tallied into two categories of either “more important” or “less important.” Mean scores will be used to determine the most important and least important factors. Univariate analysis with χ^2^ tests will then be used to determine if any demographic relations exist between what PROs participants find to be more or less important. Additionally, the Wilcoxon ranked sign test and 2-tailed ANOVA will be used to compare the differences between the patients’ answers in their first and second survey responses.

Data from the survey for the surgeons will also be analyzed with SPSS. Mean scores will be tabulated for the general surgeons’ responses to the survey. For the general surgeons’ interviews, the results will be transcribed verbatim and analyzed with thematic synthesis [24]. The qualitative data will explain the overall view that surgeons have regarding PRO collection and the quantitative data will be analyzed in conjunction to illustrate the specific PROs that the surgeons view as important. The patient and surgeon data will also be integrated by comparing the views that both stakeholders have as indicated in the PROM surveys.

### Ethical Considerations

This study has been approved by the Clinical Research and Ethics Committee of the Cork Teaching Hospitals (ECM 01/2023 PUB). The study poses a low risk to patients and does not interfere with any of the treatments that the patients will receive. Data will be stored in line with University College Cork standards and requirements.

A standardized consent form and patient information leaflet will be given to the patients, and they will be briefed on the project through information presented on the survey before they begin answering questions. Patients’ involvement will be fully voluntary and they will be given the option to withdraw from participation at any time. The researchers’ contact information will also be given to the patients so that they have any questions answered that might come up throughout the study. Only the email addresses of the patients will be collected, as patients’ identifiers so that the patients can be followed up with. The data will be pseudonymized as a result. Data will be kept on University College Cork servers for 10 years, excluding the email addresses, which will be used for following up with patients and will be immediately deleted after study completion. Patients will not be compensated for participating in this study.

### Outputs and Dissemination

The study results will be disseminated through international peer-reviewed research journals and local conference presentations.

## Results

Data collection began on February 14, 2023. As of February 12, 2024, 71 of 80 patients have been given the presurvey. The follow-up will begin once 80 patients have completed the presurvey. This is expected to be completed in March 2024 and the follow-up is expected to conclude in March 2025. General surgeons will then complete their surveys and interviews in March and April 2025. The overall expected completion date of the study is in April 2025.

## Discussion

### Study Significance and Strengths

This study will convey the perspective of participants regarding which PROs are most important for them after LCs. Based on prior research, we expect the technical skill and experience level of the surgeon, long-term quality of life, patient involvement in decision-making, communication skills of the surgeon, cleanliness of the ward environment, and standards of nursing care to be the most important factors for the patients [10]. Furthermore, this study will indicate the acceptability and perceived value of participants regarding electronic PRO surveys, which is a topic that is relatively more limited in the literature in the context of LCs. The study has well-defined inclusion and exclusion criteria, with all patients being given the survey less than 48 hours after their surgery and only elective cases being included. This will help make the sample more homogenous, as patients undergoing emergency surgery might have different perspectives on what is important for them in comparison to those having elective surgery [11].

Furthermore, the long-term follow-up of 1 year will assist in better understanding the views of patients undergoing LC. The distribution of the survey to postoperative patients will ensure that the patients already had the experience of having an LC and can reflect upon it when answering the survey. This perspective would not be achievable with patients in a preoperative setting. The use of a 50-item survey also ensures that several PROs and factors will be analyzed, thereby offering a broad view of patients’ perspectives on PROs. Moreover, the integration of the perspectives of surgeons regarding the study may help elucidate improved procedures when following up with patients.

### Limitations

As a result of this being a feasibility study, there are several associated limitations. Primarily, the small sample size of 80 participants will limit the generalizability of the study. Moreover, the use of convenience sampling also limits the external validity and generalizability of the results. It has been shown that convenience sampling has greater validity when the sample is more homogenous with respect to the participants’ sociodemographic characteristics [31]. However, making the sample completely homogenous in this study would result in an inadequate sample size. Thus, this bias will be mitigated through statistical analyses exploring differences in the factors that are deemed important according to the participants’ demographic characteristics. Another significant bias associated with using convenience sampling is that the participants accepting to partake in the study could be more likely to find perceived value in completing the electronic survey in comparison to those who declined to participate. These biases will be mitigated through the use of rigid inclusion and exclusion criteria, ensuring that the sample is as homogenous as possible.

Another limitation is the use of a 4-point Likert scale. Likert scales ideally have between 4 and 7 options, although 4-point scales might commit participants to give answers they may not wish to give [32].

Although only including patients undergoing elective surgery will aid in further defining the inclusion and exclusion criteria, this restriction will also limit the generalizability of this study, as patients undergoing emergency LC will not be represented.

In addition, recruiting patients within 48 hours of their LC means that their postoperative condition could influence their answers. The survey also contains mild medical jargon (eg, “bile leak”) that might be confusing for participants.

There is no electronic literacy scale for the participants involved. Incorporation of such a scale would have improved the interpretation of the results by demonstrating whether the ability to use technology impacts the participants’ views on electronic surveys.

The lack of direct comparison with paper surveys is another potential limitation, as this prevents a comprehensive interpretation of the benefits of electronic surveys over paper surveys. Although this study addresses the difficulty that patients might have in using electronic devices, it does not address other major barriers to electronic PROs, such as inadequate information technology infrastructure and the time needed to complete the survey [17].

The survey from Stover et al [28] may also be biased toward favorable responses. The reason for this is that the study aimed to maintain a neutral response option, while also having only 4 options to be consistent with the other survey questions. For this reason, there is only one “negative” option.

Finally, the follow-up will also be very challenging due to the small sample size. A prior study exploring email-based PRO systems following hand surgery indicated that this approach may be effective [33], although only 28% of participants completed the follow-up PRO survey 1 year after their enrollment in the study. Despite the lack of valid generalizability of the email follow-up data in this study, the results will convey the feasibility of using email to distribute PRO surveys and help guide future studies with more robust samples.

### Conclusions

Overall, this study will investigate the potential of electronic PRO collection to offer value for patients and general surgeons. This approach will ensure that patient care is investigated in a multifaceted way, offering patient-centric guidance to surgeons in their approach to care. The results will illustrate any differing views that surgeons and patients may have in relation to what is important for the patient’s care, allowing for further exploration of these differences. This study will thus guide future research to further analyze any potential uses of PROs and the value that electronic systems can provide for PRO collection.

## Appendix

Multimedia Appendix 1 Summary of all data collection points for the patient participants.

Multimedia Appendix 2 Survey for patient participants.

Multimedia Appendix 3 Interview consent sheet for general surgeons.

Multimedia Appendix 4 Procedure for interviews with general surgeons.

Multimedia Appendix 5 Survey for general surgeons.

## Abbreviations

LC: laparoscopic cholecystectomy
PREM: patient-reported experience measure
PRO: patient-reported outcome
PROM: patient-reported outcome measure
