# Supplementary material for: Acceptability, Perceptions, and Experiences Regarding Electronic Patient-Reported Outcomes After Laparoscopic Cholecystectomy: Protocol for a Mixed Methods Feasibility Study
Source: JMIR Res Protoc. 2024 Aug 19;13:e57344. doi: 10.2196/57344 (PMC11369529; doi:10.2196/57344)
Supplement: Multimedia Appendix 1 [file resprot_v13i1e57344_app1.doc]

**
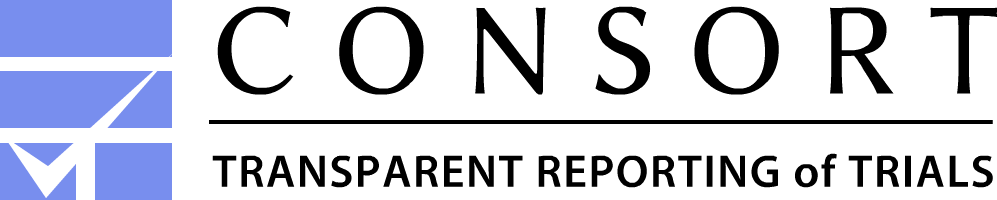
**

**Given Survey**

**Analysis**

**Follow-Up**

**Enrollment**

Assessed for eligibility

Excluded

♦  Not meeting inclusion criteria

♦  Declined to participate

♦  Other reasons

Data analysed using SPSS

Given post-survey 12 months later

Given pre-survey for the first data collection point

Drop off
