# Supplementary material for: Acceptability, Perceptions, and Experiences Regarding Electronic Patient-Reported Outcomes After Laparoscopic Cholecystectomy: Protocol for a Mixed Methods Feasibility Study
Source: JMIR Res Protoc. 2024 Aug 19;13:e57344. doi: 10.2196/57344 (PMC11369529; doi:10.2196/57344)
Supplement: Multimedia Appendix 3 [file resprot_v13i1e57344_app3.docx]

**Interview Information Sheet**

Thank you for considering participating in this research project. The purpose of this document is to explain to you what the work is about and what your participation would involve, so as to enable you to make an informed choice.

The purpose of this study to analyse patient reported outcomes after laparoscopic cholecystectomies and will involve questions regarding whether patient reported outcomes collection can improve patient care. Should you choose to participate, you will be asked to take part in a one-to-one interview with a member of the research team. This interview will be audio-recorded and will be expected to take 30min to complete.

Participation in this study is completely voluntary. There is no obligation to participate, and should you choose to do so you can refuse to answer specific questions, or decide to withdraw from the interview. Once the interview has been concluded, you can choose to withdraw your details at any time in the subsequent two weeks.

All of the information you provide will be kept confidential and anonymous, and will be available only to the research team The only exception is where information is disclosed which indicates that there is a serious risk to you or to others. Once the interview is completed, the recording will immediately be transferred to an encrypted laptop and wiped from the recording device. The interview will then be transcribed by the researcher, and all identifying information will be removed. Once this is done, the audio-recording will also be deleted and only the anonymized transcript will remain. This will be stored on the University College Cork OneDrive system and subsequently on the UCC server. The data will be stored for 10 years. The information you provide may contribute to research publications and/or conference presentations. I will debrief you afterwards and answer any questions you may have.

We do not anticipate any negative outcomes from participating in this study. Should you have any concerns arising from participating in the research, or should it raise any issues for you, the contact details for support services provided below may be of assistance.

This study has obtained ethical approval from the UCC Clinical Research and Ethics Committee.

If you have a concern about how we have handled your personal data, you are entitled to this raise this with the Data Protection Commission.

<https://www.dataprotection.ie/>

If you have any queries about this research, you can contact me at [119100174@umail.ucc.ie](mailto:119100174@umail.ucc.ie) or the principal investigator at [john.odonoghue@ucc.ie](mailto:john.odonoghue@ucc.ie)

The Data Controller for this study is John O’Donoghue ([john.odonoghue@ucc.ie](mailto:john.odonoghue@ucc.ie))

If you agree to take part in this study, please sign the consent form overleaf.

**Consent Form**

I………………………………………agree to participate in Kareem Choucair’s research study.

The purpose and nature of the study has been explained to me in writing.

I am participating voluntarily.

I give permission for my interview with Kareem Choucair to be audio-recorded.

I understand that I can withdraw from the study, without repercussions, at any time, whether before it starts or while I am participating.

I understand that I can withdraw permission to use the data within two weeks of the interview, in which case the material will be deleted.

I understand that anonymity will be ensured in the write-up by disguising my identity.

I understand that disguised extracts from my interview may be quoted in the thesis and any subsequent publications if I give permission below:

(Please tick one box:)

I agree to quotation/publication of extracts from my interview ☐

I do not agree to quotation/publication of extracts from my interview ☐

Signed: ……………………………………. Date: ………………..

PRINT NAME: …………………………………….
