# Supplementary material for: Acceptability, Perceptions, and Experiences Regarding Electronic Patient-Reported Outcomes After Laparoscopic Cholecystectomy: Protocol for a Mixed Methods Feasibility Study
Source: JMIR Res Protoc. 2024 Aug 19;13:e57344. doi: 10.2196/57344 (PMC11369529; doi:10.2196/57344)
Supplement: Multimedia Appendix 4 [file resprot_v13i1e57344_app4.docx]

I am going to use the term PROMs throughout the interview. This stands for Patient-reported outcome measures which are questionnaires that assess patients’ health including: symptoms, function, well-being, health-related quality of life (HRQOL) and other health related constructs.

- Would you mind if I record this interview? Anything we discuss will remain confidential and your identity will remain anonymous in any publications or reports. Finally, you can stop the interview at any point if you wish. Do you have any questions for me before we get started?
- Sign consent and give copy

**Background**

- Firstly, could you tell me about your experience with the use of PROMs?
- Have you (or the hospital you work in) collected PROMs before we begun this trial?
- YES
  - What measures do you collect?
  - How do you use this data (dissemination: reports, meetings)?
  - How do you think this information should be used?
- No
  - Can you explain to me any QI initiative in which you involved the patient?
  - What is your experience of QI initiatives in the hospitals you work in?

**Attitudes**

- What are your views on the collection and use of PROMs?
- In particular, what are your opinions on the use of PROMs as a QI tool?
- How would you feel if this data was used :
- As a clinical governance tool in the hospital(s) you work in?
- To inform patient choice by publically reporting the data?
- To inform purchasers decisions?
- To link payment to results?
- How do you think PROMs should be used?
- Would you like to receive regular feedback reports?
