# Supplementary material for: Acceptability, Perceptions, and Experiences Regarding Electronic Patient-Reported Outcomes After Laparoscopic Cholecystectomy: Protocol for a Mixed Methods Feasibility Study
Source: JMIR Res Protoc. 2024 Aug 19;13:e57344. doi: 10.2196/57344 (PMC11369529; doi:10.2196/57344)
Supplement: Supplementary file 4 [file resprot_v13i1e57344_app2.docx]

DS-PRO Study - 2

Start of Block: Default Question Block

Q2 Thank you for considering participating in this research project. The purpose of this section is to explain to you what the work is about and what your participation would involve, so as to enable you to make an informed choice. The purpose of this study is to analyse patient outcomes after laparoscopic cholecystectomies and will involve questions regarding the psychological and physical effects that the surgery had on patients. Participation in this study is completely voluntary. There is no obligation to participate, and should you choose to do so you can refuse to answer specific questions, or decide to withdraw from the study. All information you provide will be confidential and your anonymity will be protected throughout the study. IP addresses will not be collected at any point, meaning the data you provide cannot be traced back to you.

You maintain the right to withdraw from the study at any stage up to the point of data submission. 

At this point your data will be collated with that of other participants and can no longer be retracted. The anonymous data will be stored on the University College Cork OneDrive system and subsequently on the UCC server. The data will be stored for a minimum of 10 years, but your email will be removed from our servers immediately after the second survey distribution is complete in approximately 12 months. You will be asked for your email at the end of this survey, through a separate and anonymised link. This ensures that your email can not be matched with your response, maintaining the anonymity and confidentiality of your responses. The information you provide may contribute to research publications and/or conference presentations. It will also help inform surgeons regarding the best practices that they must uphold to ensure that laparoscopic cholecystectomies are most beneficial for patients. I will debrief you afterwards and answer any questions you may have. We do not anticipate any negative outcomes from participating in this study. Some of the questions may be personal in nature and should you not wish to answer any of the questions you may do so. Should you experience any concerns arising from participating in the research, or should it raise any issues for you, the contact details for support services provided below may be of assistance. This study has obtained ethical approval from the UCC Clinical Research Ethics Committee of the Cork Teaching Hospitals. The survey has an expected completion time of 15 minutes. You will be asked to complete another survey in 12 months.

If you have any queries about this research, you can contact me at 119100174@umail.ucc.ie or contact the principal investigator involved in this project through the email john.odonoghue@ucc.ie. 

 If you have a concern about how we have handled your personal data, you are entitled to this raise this with the Data Protection Commission. https://www.dataprotection.ie/. “A personal data breach occurs when the data is accessed, disclosed, altered, lost or destroyed in contravention of an organisation’s obligation to keep personal data in its possession safe and secure” https://www.dataprotection.ie/ UCC'S Data Protection Officer (DPO) is Catriona O'Sullivan, Information Compliance Manager, University College Cork, 4 Carrigside, College Road, Cork, Ireland. Telephone: +353 (0)21 4903949* Email: gdpr@ucc.ie The Data Controller for this study is Dr. John O'Donoghue (john.odonoghue@ucc.ie). If you have a complaint about how this research was conducted please contact in writing: The Ethics Committee, School of Applied Psychology, University College Cork, Cork. If you have a complaint about how this research was conducted please contact in writing: The Ethics Committee, School of Applied Psychology, University College Cork, Cork If you agree to take part in this study, please complete the consent form overleaf.

Q4  

|  | Click here to Confirm (1) |
| --- | --- |
| Click to consent to participate in this study. (1) |  |

End of Block: Default Question Block

Start of Block: Block 8

Q68 
**Data Protection Notice**

At University College Cork, we treat your privacy seriously. Any personal data which you provide to the University will be treated with the highest standards of security and confidentiality, in accordance with Irish and European Data Protection legislation. This notice sets out details of the information that we collect, how we process it and who we share it with. It also explains your rights under data protection law in relation to our processing of your data.

**Who we are**
Throughout this Notice, “we”, “us” and “our” refers to University College Cork, as study sponsor. For more information about us, please refer to our website: www.ucc.ie

**How we will use your personal data**
By participating in the study, information from you (also called “personal data”) will be collected for the study purposes mentioned in the Participant Information Leaflet above. This personal data may include, for example:

 −      information that directly identifies you (such as your name, and your year of birth)
 −      your gender, ethnic and racial background
 −      information on your health and medical condition including your medical history
 −      your treatments and your response to treatments
 −      information contained in your blood samples and the results after analysis

 Personal data collected at any time during the study will be kept strictly confidential. To ensure confidentiality, the data generated during the study is coded with a number that will identify you in the study. Any information that leaves the clinical site will be labelled with your code instead of your name. 

Data that directly identifies you (uncoded data) is stored securely by the Chief Investigator.  A list or ‘key’ linking your study number to your name will be kept stored securely (locked cupboard in a room with restricted access) by the Chief Investigator for 10 years & destroyed thereafter. The study team who access to your uncoded data are subject to professional secrecy and confidentiality.

**Who will access my personal data?**
Your uncoded data will only be accessible to the study investigator and study staff. Results of the study will be provided to the Clinical Research Ethics Committee of the Cork Teaching Hospitals (CREC) in compliance with national and international regulations on clinical studies.

**The purpose and legal basis for collecting your data**
Any personal data you provide to us during the course of this study will be processed fairly and lawfully.  Signing the Informed Consent Form means that your personal data will be used for the purposes outlined in the Participant Information Leaflet.
Personal data collected during this study and the results of the study may be presented for scientific purposes. However, you will never be identified individually during these presentations. Your identity will not be revealed in any reports or publications.  The clinical site and the investigator will use your personal data within the scope defined above.  
The General Data Protection Regulation allows us to process your data because you have provided your consent. You are entitled to withdraw your consent at any time. 

**How long we will keep your data**
The personal data collected in the study will be kept for a period of at least 5 years after the end of the study. Thereafter, they may be stored for a further period of time for legal reasons (e.g. revised retention obligations), or more if required by law.

**Your rights**
You have various rights under data protection law, subject to certain exemptions, in connection with our processing of your personal data, including the right:

•	to find out if we use your personal data, access your personal data and receive copies of your personal data.
• to have inaccurate/incomplete information corrected and updated.
•	in certain circumstances, to have your details deleted from systems that we use to process your personal data or have the use of your personal data restricted in certain ways.
• to object to certain processing of your data by UCC. 
•	to exercise your right to data portability where applicable (i.e. obtain a copy of your personal data in a commonly used electronic form. 
•	to withdraw your consent to the processing of your data at any time without giving a reason by notifying your decision to the investigator. This will not affect the lawfulness of processing data about you based on your consent before the withdrawal. If you withdraw your consent for data processing, your participation in the study stops and no further data will be collected from you. Your study physician will present you the options you have concerning your personal data. 
•	Along with study withdrawal, you have the right to request the deletion of data about you if your data are no longer necessary for the purposes of processing or there is no other legal ground for their further processing.
If you wish to exercise any of these rights, please address your request to the study physician or the Information Compliance Manager, University College Cork (details below). 

**Questions or Complaints**
If you have any queries in relation to this study, please contact the Chief Investigator (the doctor in charge of the study which is detailed at the top of the Participant Information Leaflet). 

 If you have any complaints in connection with our processing of your personal data, you can contact UCC’s Information Compliance Manager: Information Compliance Manager, Office of Corporate & Legal Affairs, University College Cork, Western Road, Cork E: foi@ucc.ie Tel: +353 21 4903949 

You also have the right to lodge a complaint with the Data Protection Commission if you are unhappy with our processing of your personal data. Details of how to lodge a complaint can be found on the Data Protection Commission’s website (www.dataprotection.ie), or by telephoning 1890 252 231.

Q69  

|  | Click here to Confirm (1) |
| --- | --- |
| Click to consent to participate in this study. (1) |  |

End of Block: Block 8

Start of Block: Block 1

Q5 What is your age range?

- 18-29 years  (1) 

- 30-39 years  (2) 

- 40-49 years  (3) 

- 50-59 years  (4) 

- 60-69 years  (5) 

- 70-79 years  (6) 

- 80-89 years  (7) 

- 90+  (8) 

Q6 What is your gender?

- Male  (1) 

- Female  (2) 

- Non-binary / third gender  (3) 

- Prefer not to say  (4) 

Q7 What is your education level?

- Primary school  (1) 

- Secondary school  (2) 

- Diploma (leaving cert or equivalent)  (3) 

- Graduate degree  (4) 

- Post-graduate degree  (5) 

Q8 What is your marital status?

- Single, never married  (1) 

- Married or partner  (2) 

- Widowed  (3) 

- Divorced or separated  (4) 

- Prefer not to disclose  (5) 

Q9 What is your employment status?

- Unemployed  (1) 

- Self-employed  (2) 

- Working/employed  (3) 

- Student  (4) 

- Retired  (5) 

- Homemaker  (6) 

- Unable to work  (7) 

End of Block: Block 1

Start of Block: Block 2

Q11 Do you smoke?

- Yes  (1) 

- Never  (2) 

- Used to, now stopped  (3) 

Q12 Do you exercise?

- Regular  (1) 

- Occasional  (2) 

- Never  (3) 

Q13 How important to you is waiting time for the surgery

- You prefer as soon as possible  (1) 

- You prefer as soon as doctor can do  (2) 

- Waiting time is not important to you  (3) 

Q14 After consulting the surgeon for the first time, how long had you to wait for the surgery to be scheduled?

- Less than 4 weeks  (1) 

- 4-8 weeks  (2) 

- 8-12 weeks  (3) 

- More than 12 weeks  (4) 

Q15 How important is it for you to stay under the care of the same consultant doctor (senior doctor)?

- Very Important  (1) 

- Important  (2) 

- Less important  (3) 

- Least important  (4) 

Q16 How good was your pain control after surgery?

- No pain at all  (1) 

- Some pain, but controlled and was within expectations  (2) 

- More than expected pain  (3) 

- Horrifying and terrifying experience, wish had not undergone the surgery  (4) 

End of Block: Block 2

Start of Block: Block 3

Q17 How important to you is each of the following aspect of your procedure?

Q18 Duration of the procedure

- Very important  (1) 

- Important  (2) 

- Less important  (3) 

- Least important  (4) 

Q19 Successful key hole surgery

- Very important  (1) 

- Important  (2) 

- Less important  (3) 

- Least important  (4) 

Q20 Amount of blood loss

- Very important  (1) 

- Important  (2) 

- Less important  (3) 

- Least important  (4) 

Q21 Overall risk of complications

- Very important  (1) 

- Important  (2) 

- Less important  (3) 

- Least important  (4) 

Q22 Risk of severe complications

- Very important  (1) 

- Important  (2) 

- Less important  (3) 

- Least important  (4) 

Q23 Risk of minor complications

- Very important  (1) 

- Important  (2) 

- Less important  (3) 

- Least important  (4) 

Q24 Risk of bile duct injury

- Very important  (1) 

- Important  (2) 

- Less important  (3) 

- Least important  (4) 

Q25 Risk of bile leak

- Very important  (1) 

- Important  (2) 

- Less important  (3) 

- Least important  (4) 

Q26 Risk of retained stone within

- Very important  (1) 

- Important  (2) 

- Less important  (3) 

- Least important  (4) 

Q27 Risk of anaesthesia

- Very important  (1) 

- Important  (2) 

- Less important  (3) 

- Least important  (4) 

End of Block: Block 3

Start of Block: Block 4

Q28 How important to you are the following aspects of the recovery process?

Q29 Going home on the same day

- Very important  (1) 

- Important  (2) 

- Less important  (3) 

- Least important  (4) 

Q30 Going home the next day

- Very important  (1) 

- Important  (2) 

- Less important  (3) 

- Least important  (4) 

Q31 Short time to recovery of normal function eg. doing normal daily activities

- Very important  (1) 

- Important  (2) 

- Less important  (3) 

- Least important  (4) 

Q32 Shorter period of medical leave

- Very important  (1) 

- Important  (2) 

- Less important  (3) 

- Least important  (4) 

Q33 Shorter time before returning to normal diet

- Very important  (1) 

- Important  (2) 

- Less important  (3) 

- Least important  (4) 

Q34 Cost of the hospital stay/procedure

- Very important  (1) 

- Important  (2) 

- Less important  (3) 

- Least important  (4) 

End of Block: Block 4

Start of Block: Block 5

Q40 How important to you are the following elements of the overall hospital experience?

Q41 Your involvement in the decision making

- Very important  (1) 

- Important  (2) 

- Less important  (3) 

- Least important  (4) 

Q42 Communication skill of the doctor/surgeon

- Very important  (1) 

- Important  (2) 

- Less important  (3) 

- Least important  (4) 

Q43 Technical/surgical skills of the doctor/surgeon

- Very important  (1) 

- Important  (2) 

- Less important  (3) 

- Least important  (4) 

Q44 Dietary advice before and after the doctor/surgeon

- Very important  (1) 

- Important  (2) 

- Less important  (3) 

- Least important  (4) 

Q45 Reputation of the doctor/surgeon performing the surgery

- Very important  (1) 

- Important  (2) 

- Less important  (3) 

- Least important  (4) 

Q46 Cleanliness of the ward/theatre area

- Very important  (1) 

- Important  (2) 

- Less important  (3) 

- Least important  (4) 

Q47 Standards of the nursing care

- Very important  (1) 

- Important  (2) 

- Less important  (3) 

- Least important  (4) 

Q48 Opinion of family and friends about the hospital

- Very important  (1) 

- Important  (2) 

- Less important  (3) 

- Least important  (4) 

Q49 Hospital provides you with contact details about the ward on discharge

- Very important  (1) 

- Important  (2) 

- Less important  (3) 

- Least important  (4) 

Q50 Standards of hospital food

- Very important  (1) 

- Important  (2) 

- Less important  (3) 

- Least important  (4) 

End of Block: Block 5

Start of Block: Block 6

Q53 How important to you are the following aspects of long-term outcomes?

Q51 Cosmetic appearance of the scar

- Very important  (1) 

- Important  (2) 

- Less important  (3) 

- Least important  (4) 

Q54 Residual abdominal symptoms after the procedure

- Very important  (1) 

- Important  (2) 

- Less important  (3) 

- Least important  (4) 

Q56 Overall satisfaction with the procedure

- Very important  (1) 

- Important  (2) 

- Less important  (3) 

- Least important  (4) 

Q55 Quality of life after surgery

- Very important  (1) 

- Important  (2) 

- Less important  (3) 

- Least important  (4) 

End of Block: Block 6

Start of Block: Block 7

Q60 How would you describe seeing questions on your device?

- Easy  (1) 

- Somewhat easy  (2) 

- Neither difficult nor easy  (3) 

- Difficult  (4) 

Q61 How would you describe using your device to answer questions

- Easy  (1) 

- Somewhat easy  (2) 

- Neither difficult nor easy  (3) 

- Difficult  (4) 

Q62 How would you describe answering questions about your symptoms and health?

- Easy  (1) 

- Somewhat easy  (2) 

- Neither difficult nor easy  (3) 

- Difficult  (4) 

Q64 In general, how satisfied were you with using your device to report your symptoms?

- Satisfied  (1) 

- Somewhat satisfied  (2) 

- Neither satisfied nor unsatisfied  (3) 

- Unsatisfied  (4) 

Q65 How helpful was the symptom survey to remind you of symptoms you experienced in the last 7 days?

- Helpful  (1) 

- Somewhat helpful  (2) 

- Neither helpful nor unhelpful  (3) 

- Unhelpful  (4) 

Q67 How helpful was the symptom survey for talking to your doctor/ nurse about symptoms you experienced?

- Helpful  (1) 

- Somewhat helpful  (2) 

- Neither helpful nor unhelpful  (3) 

- Unhelpful  (4) 

End of Block: Block 7

Start of Block: Block 9

Q70 Insert your email here, so that we can send you the follow up survey

________________________________________________________________

End of Block: Block 9
